# Coverage Hole Recovery in Hybrid Sensor Networks Based on Key Perceptual Intersections for Emergency Communications

**DOI:** 10.3390/s25134217

**Published:** 2025-07-06

**Authors:** He Li, Shixian Sun, Chuang Dong, Qinglei Qi, Cong Zhao, Zufeng Fu, Peng Yu, Jiajia Liu

**Affiliations:** 1 Henan Intelligent Emergency Service and Security Engineering Research Center, Nanyang Normal University, Nanyang 473061, China; lihe@nynu.edu.cn (H.L.); sunshixian2000@163.com (S.S.); goodyt@163.com (Z.F.); liujiajia20041225@163.com (J.L.); 2 Collaborative Innovation Center of Intelligent Explosion-Proof Equipment, Henan Province, Nanyang Normal University, Nanyang, 473061, China; charon_55@163.com (C.D.); qiqinglei@nynu.edu.cn (Q.Q.); 3 The State Key Laboratory of Networking and Switching Technology, Beijing University of Posts and Telecommunications, Beijing 100876, China; yupeng@bupt.edu.cn

**Keywords:** wireless sensor network, coverage holes detection, coverage holes recovery, key perception intersection, PSO

## Abstract

Wireless sensor networks (WSNs) have found extensive applications in a variety of fields, including military surveillance, wildlife monitoring, industrial process monitoring, and more. The gradual energy depletion of sensor nodes with limited battery energy leads to the dysfunction of some of the nodes, thus creating coverage holes in the monitored area. Coverage holes can cause the network to fail to deliver high-quality data and can also affect network performance and the quality of service. Therefore, the detection and recovery of coverage holes are major issues in WSNs. In response to these issues, we propose a method for detecting and recovering coverage holes in wireless sensor networks. This method first divides the network into equally sized units, and then selects a representative node for each unit based on two conditions, called an agent. Then, the percentage of each unit covered by nodes can be accurately calculated and holes can be detected. Finally, the holes are recovered using the average of the key perceptual intersections as the initial value of the global optimal point of the particle swarm optimization algorithm. Simulation experiments show that the algorithm proposed in this paper reduces network energy consumption by 6.68%, decreases the distance traveled by mobile nodes by 8.51%, and increases the percentage of network hole recovery by 2.16%, compared with other algorithms.

## 1. Introduction

Wireless sensor networks (WSNs) represent a novel paradigm in computing and networking, characterized as a system consisting of minute, expensive, and highly intelligent devices, commonly referred to as sensor nodes [1,2]. A wireless sensor network (WSN) is a structure composed of numerous sensor nodes that communicate wirelessly. It is extensively utilized in the industrial field for detecting and monitoring key target areas, such as urban tracking and observation [3], environmental analysis [4], strategic surveillance [5], mobile target tracking [6], and smart home automation [7]. The deployment environment of a WSN is highly variable, presenting numerous challenges. Sensor nodes may be deployed in areas where the environment is harsh and manpower cannot work, and the death of a sensor node may be caused by its own factors such as the exhaustion of energy required by the node and hardware failure, as well as environmental factors such as inclement weather and external attacks. Once a node loses its sensing capability, it is unable to complete the information collection and reporting tasks within its original sensing range. The network area that has been well covered and monitored is damaged, and a network coverage hole is formed [8]. The coverage hole will impair data transmission and disconnect the connected network, resulting in unobservable critical events or inaccurate data, greatly reducing the integrity of the WSN in collecting information from the covered area, and degrading and destroying the functionality of the entire network. After a disaster, to ensure the communication quality and meet various rescue communication needs, it is necessary to restore the composite communication of the whole area in a high-efficiency way. Therefore, sensor nodes are deployed to complete the full coverage of the disaster area to achieve the recovery of basic communications.

When the network is divided into so many units and several units are detected to have coverage holes, then we need to select suitable mobile nodes to fix the coverage holes. The algorithm in this paper has the option of moving nodes to fix holes, but its fitness function is slightly simplistic and has a lot of room for improvement [9]. In this paper, the fitness function not only considers the distance of the hole from the BS, the coverage of the hole, but also considers the overlap rate with other nodes when the mobile node dispatches over.

The term key perception intersection point is abbreviated as KIP. A perception intersection point refers to the intersection between the sensing circle of a node in the surveillance area and its neighboring nodes, or the intersection between that node and the boundary of the detection area. A perception intersection is termed a key perception intersection if it is not covered by any other neighboring nodes [10]. If the KIP of a unit is determined, the centroid of the coverage hole can be approximated. If the centroid of the coverage hole is known, mobile nodes can be directly dispatched to fix the hole [11]. However, the single use of a KIP to repair the hole has many issues, such as the peculiar shape of the hole, a too high overlap rate with other nodes, etc. To overcome these problems, we combine the KIP with PSO, and consider the overlap rate of mobile nodes with other nodes when setting the fitness function, which achieves good results.

To address these issues and reduce the energy consumption and the mobility distance of the mobile nodes, this paper proposes a method for hole detection and recovery based on a key perception intersection (partial research results were published in the IEEE International Symposium on Parallel and Distributed Processing with Applications ISPA 2024). In this method, the network is firstly united. Then, the percentage of each unit covered by nodes is accurately calculated and holes are detected. Finally, the hole is recovered by using the average of the key perception intersections as the initial value of the global optimal point of the PSO. The key contributions presented in this paper can be condensed as:

(1) The paper introduces a network unit formulation rooted in network density, a metric derived from the interplay between network scale and the count of nodes. As network density escalates, so does the proliferation of units, fostering the enhanced load distribution and the streamlined management of network nodes.

(2) The entire network environment is divided into units, each discretized into k*k pixel points. Each unit has an information table with the number of rows equal to the number of pixels covered by the unit and the number of columns in the table equal to the number of nodes in the unit. The method can accurately calculate the percentage of each unit covered by nodes and can accurately detect the location of holes. The accurate identification of holes can improve the recovery efficiency, coverage, and quality of service of the network.

(3) Using mobile nodes to recover holes improves coverage. Recovering holes by using the average value of the key perceived intersection as the initial value of the global optimal point of PSO reduces the distance traveled by the mobile nodes and improves the overall coverage of the nodes.

The rest of this article is structured as follows: Section 2 reviews the literature pertinent to our work. Section 3 describes network scenarios and several models. Section 4 provides a detailed description of the proposed method. Section 5 presents the simulation results and evaluation of the proposed method. Finally, some conclusions are given in Section 6.

## 2. Related Work

In recent times, numerous methods have been developed to tackle the problem of coverage holes in WSNs. These approaches can generally be divided into three categories according to the type of network: static sensor networks, mobile sensor networks, and hybrid sensor networks.

### 2.1. Static Sensor Networks

Since nodes are not mobile, the main consideration is the deployment strategy of the nodes, which is more like network optimization. By scheduling the monitoring area to be fully covered by sensor nodes simultaneously, the overlapping areas between nodes can be minimized, thereby maximizing the overall coverage of the network. The focus of the static WSN solution is to find the smallest set of sensor nodes that can fully monitor the covered area, utilizing an effective clustering method with a sensor activation scheduling strategy, and combining it to reduce the energy consumption for network deployment and improve the energy utilization, thus extending the network lifecycle and maintaining the coverage [12]. A sensor deployment method based on the bee clustering algorithm schedules sensor nodes to achieve the longest lifecycle while minimizing the overlap region and the number of nodes used at the time of deployment [13]. Paper [14] proposes an effective genetic algorithm, MIGA, for the maximization of the area coverage in static wireless sensor networks with heterogeneous sensing ranges. However, this method has a relatively large computational load and longer computation time. For example, when processing the s5-09 instance, the average computation time for MIGA is about 60,000 ms, while the average computation time for ICS is about 20,000 milliseconds. Its computational complexity is also too high (O(n^3^logn)). Paper [15] proposed a distributed energy efficiency optimization method based on the firefly algorithm, which divides the network geography into grids and balances the network energy consumption. However, the applicability and efficiency in dynamic and complex network environments are relatively low. Hole remediation can be achieved by activating the neighbor node with the highest number of sensor nodes at the hole edge. But this leads to high node-to-node overlap [16]. The complexity of this algorithm is high and the redundancy of the nodes also has a significant impact on the network. The algorithm proposed in this paper limits the redundant coverage of critical targets and improves network lifetime by prioritizing the sensors that cover critical targets and have high remaining battery life [17]. The algorithm WEMER in this paper merges the wedge regions dynamically by forming sectors and wedges during the initialization phase of the network and merging the wedges dynamically when energy voids are formed. It also effectively balances the energy consumption in the network and prolongs the lifetime of the network by considering the residual energy of the nodes and the distance from the relevant successor cluster head when selecting the cluster head [18]. Paper [19] calculates node positions using an improved Lyapunov optimization algorithm, performs clustering, and calculates the area of holes using an improved sand cat swarm optimization algorithm, and then detects the shape and size of holes using hybrid deep reinforcement learning technology. However, the training process of Hyb-DRL technology requires a large amount of data and time, which may result in a long model training time and difficulty in quickly adapting to dynamic changes in the network. The paper [20] uses ISGO to quantify the area covered by holes, DGO to accurately locate hole boundaries, and RGNN to select patch locations, significantly improving the efficiency of hole detection and repair. However, DGO may have shortcomings in terms of positioning accuracy, especially in complex network environments, and the accuracy of boundary identification may be challenged. Paper [21] locates holes by calculating the area of the circumscribed circle and assigns repair priorities based on hole size. However, these com-plex calculations consume a significant amount of computing resources and increase the energy consumption of sensor nodes.

### 2.2. Mobile Sensor Networks

All sensors can be moved after deployment, and when a coverage gap occurs within a network, mobile nodes can be directly dispatched to fix the coverage gap. The main concern of mobile sensor networks is the selection of appropriate nodes and their deployment to desired locations to maximize the coverage area. In paper [22], the movement of a vast quantity of sensor nodes is utilized to achieve a balanced distribution of nodes within the network and to eliminate coverage holes. In paper [23], hole detection and recovery use multiple computational geometric steps such as Voronoi diagram construction, Delaunay triangular dissection, and convex packet formation, which can lead to high computational complexity. Despite acknowledging the importance of the distance between the virtual repair node and the mobile node, as well as the energy expenditure incurred during the mobile repair operations, the paper does not fully consider the energy consumed by all of the nodes in the repair process. In this paper [24], according to the perception probability of the nodes, a node movement strategy is designed so that the nodes move to a region with low perception probability, but there is a problem with the insufficient control of the movement of the nodes in the dense region. In paper [25], we propose an algorithm that combines node mobility and adjustable sensing range to fix the coverage holes, but the nodes need to adjust their sensing range or position frequently. Although the genetic algorithm and particle swarm optimization algorithm proposed in this paper theoretically improve the coverage performance of wireless sensor networks, they suffer from the problems of difficult parameter adjustment but easily fall into local optimal solutions [26]. The approach proposed in the paper [27], while aiming to address the energy and coverage hole problems in underwater wireless sensor networks, suffers from high node energy consumption and a high dependence on initial deployment.

### 2.3. Hybrid Sensor Networks

Hybrid WSNs combine static and mobile sensor networks. Initially static sensor nodes are deployed and later when holes are found, holes are fixed by dispatching mobile nodes. In the paper [28], a self-healing coverage scheme based on fuzzy logic is proposed for randomly deployed mobile sensor networks. The scheme minimizes coverage holes by identifying uncovered sensing areas and selecting the best mobile node within moments. However, it relies on a fuzzy logic system for node selection and mobility decisions, which may increase the computational complexity and may lead to an insufficient response in rapidly changing network environments. Paper [29] balances the load by dividing the detection area into equal-sized units, while calculating node overlaps with high accuracy to schedule sensor nodes and reduce network energy consumption. However, the algorithm requires high computational resources to achieve accurate node overlap calculation and hole detection. Paper [30] proposes a dynamic clustering method based on a two-layer WSN–IoT architecture to detect coverage holes by clustering members, accurately identifying holes using the surveyor’s formula, and recovering holes using fuzzy logic. However, the detection and recovery of coverage holes using the surveyor’s formula and fuzzy logic is computationally complex and consumes more computational resources. Also, the recovery strategy proposed in the article is not applicable in some specific cases, such as when there are a lot of coverage holes in the network or when the nodes have an extreme energy imbalance. The algorithm in this paper estimates the node’s overlap with other neighboring nodes by converting the sensory range of each sensor node into a numerical matrix, and then designs a scheduling mechanism based on Q-learning. However, using Q-learning and numerical matrices to calculate node overlap increases the computational burden of sensor nodes, especially in resource-constrained environments [31].

This shows that swarm intelligence optimization algorithms are more popular and have more research value in solving coverage holes. KIP is also frequently used in coverage vulnerability detection. Combining KIP with swarm intelligence optimization algorithm is of great significance for coverage hole fixing.

## 3. System Model

### 3.1. Network Model

In WSNs, *n* static UAVs and *m* mobile UAVs are randomly deployed in a rectangular target area *O*. It is assumed that all mobile nodes are initially deployed with all of them dormant and located at the base station. In addition, the entire network is well connected regardless of the presence of coverage voids. The following assumptions are made for the network model used in this chapter to ensure the subsequent work:

**Assumption** **1.** *n static UAVs and m mobile UAVs are randomly deployed in the WSNs target area. The set of UAVs is defined as S = {S_1_, S_2_, …, S_n_, S_n+1_, …, S_n+m_}, where S_i_ = (x_i_, y_i_) (i = 1, 2, 3, …, n + m) is the position of each UAV.*

**Assumption** **2.** *Each UAV knows its location information through GPS or some location service, and each node has a unique ID number* [32].

**Assumption** **3.** *Assume that the UAV randomly deployed in the WSNs are isomorphic with a sensing radius of R_s_, a communication radius of R_c_, and both the communication range and the sensing range are circular areas centered on the node position and radiused by R_s_, R_c_, respectively. To ensure the normal transmission of data, the communication radius of the node is set equal to twice the sensing radius of the node, i.e., R_c_ = 2R_s_* [33].

**Figure 1:**
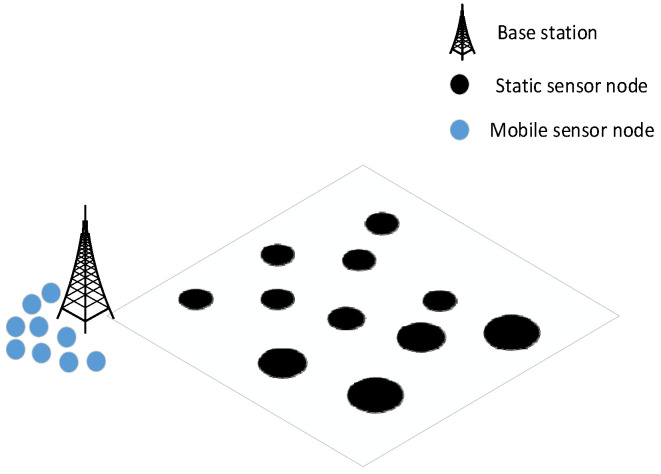
The network model.

The network model of the method in this paper is shown in Figure 1.

### 3.2. Sensing Model

In the context of WSN coverage, two primary sensing models exist: the Boolean sensing model and the probabilistic sensing model. The Boolean sensing model is also referred to as the 0–1 sensing model. Given its relative simplicity compared with the probabilistic sensing model, we will employ the Boolean sensing model in this paper, as detailed below:

(1) $$\;P_{i,j}=\left\{\begin{array}{c} 1,d_{\left(i,j\right)}\leq R_{s}\\ 0,otherwise \end{array}\right.$$

where *d*_(*i*,*j*)_ denotes the Euclidean distance between node *S_i_* and the target at point *j*, and *R_s_* is the perception radius of the node. Through (1), it is obvious that when the monitored object is within the sensing range of node *S_i_*, the probability that the monitored object is covered by effective sensing is 1. Otherwise, its probability is constant at 0.

### 3.3. Energy Model

The energy consumption model presented in paper [34] is utilized for calculating energy consumption. To ensure efficient data transmission, the node amplifies the signal during the data transmission process based on the transmission distance. Assuming the data transmission distance is *d*, the energy consumption for a node to transmit b bits of data over the link can be expressed as:

(2) $$\;E_{tx}\left(k,d\right)=\left\{\begin{array}{c} kE_{elec}+k\varepsilon_{fs}d^{2}\;if\;d<d_{0}\\ kE_{elec}+k\varepsilon_{amp}d^{4}\;if\;d\geq \;d_{0} \end{array}\right.$$

(3) $$d_{0}=\sqrt{\frac{\varepsilon_{fs}}{\varepsilon_{amp}}}$$

where $E_{elec}$ denotes the energy consumed by the node to transmit 1 bit data, $\varepsilon_{fs}$ is the free space model signal amplification factor, and $\varepsilon_{amp}$ is the multipath fading model signal amplification factor.

The energy consumed by the node to receive *k* bit data is given in the following equation:

(4) $$\;E_{rx}\left(k,d\right)=k*E_{elec}$$

### 3.4. Hole Model

**Figure 2:**
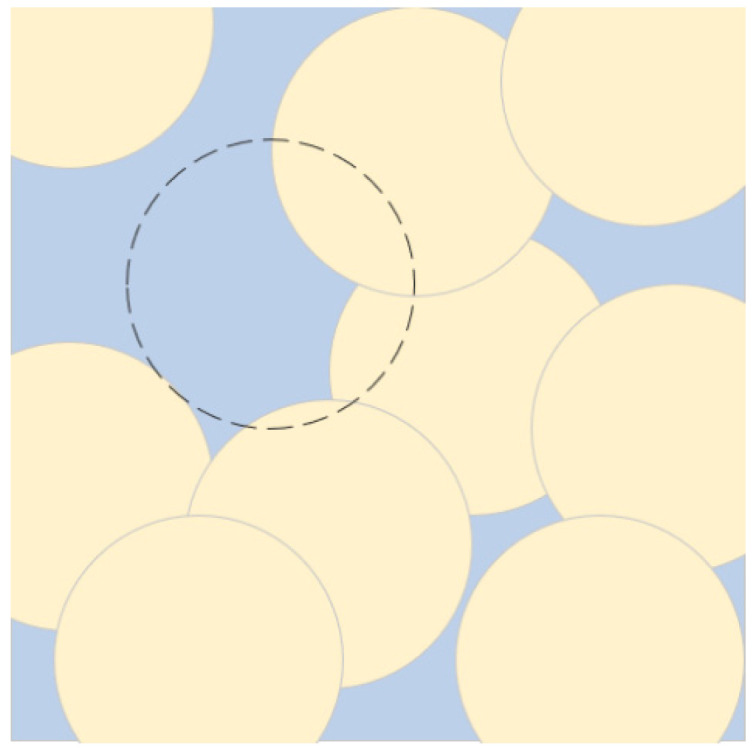
Coverage hole.

Since UAVs have limited battery energy, where the gradual depletion of energy leads to the dysfunction of some nodes, thus creating holes in the monitored area. The area covered by the node is calculated as a percentage of the area of the unit; if it is less than 90% then the unit is considered to have a coverage hole—as shown in Figure 2. For example, when the nodes at the dotted line run out of energy, resulting in the coverage area of this unit being less than 90 percent, the unit is considered to have a coverage hole.

### 3.5. Network Scenario

After a disaster, to ensure the quality of communications and to meet various rescue communication needs, it is necessary to efficiently restore communications in the entire region. As the ground is severely damaged after a disaster, the probability of user movement is very small, and at this time the user’s position can be considered relatively static. In such situations, users’ communication needs primarily include high reliability for data transmission to ensure the timely delivery of emergency information; low latency to enable rapid response to emergencies; and sufficient coverage to ensure that all disaster-affected areas can be monitored. Therefore, aerial base stations need to be established, and the deployment of UAVs should ensure full coverage of the disaster area to realize the restoration of basic communications.

**Figure 3:**
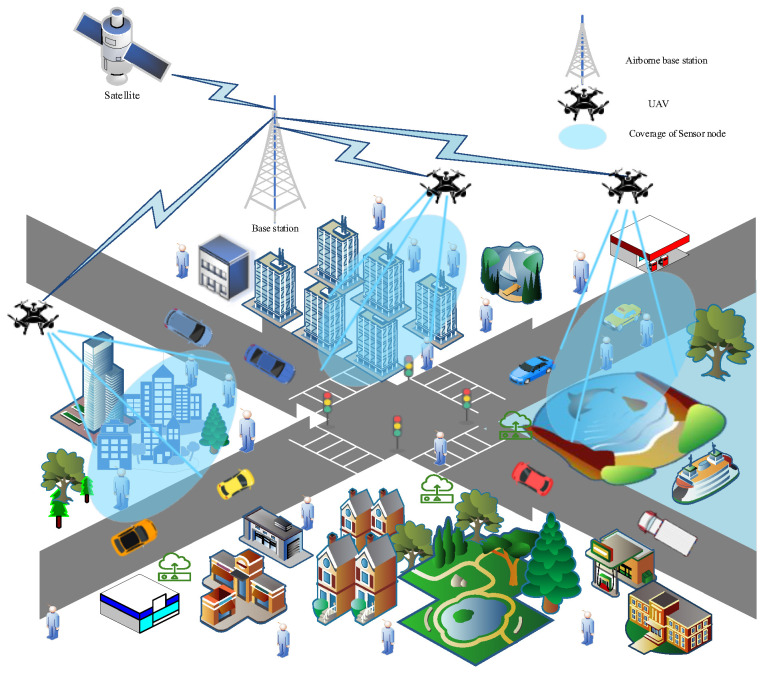
Emergency communications network scenario.

The emergency communications scenario is shown in Figure 3.

## 4. Proposed Methodology

**Figure 4:**
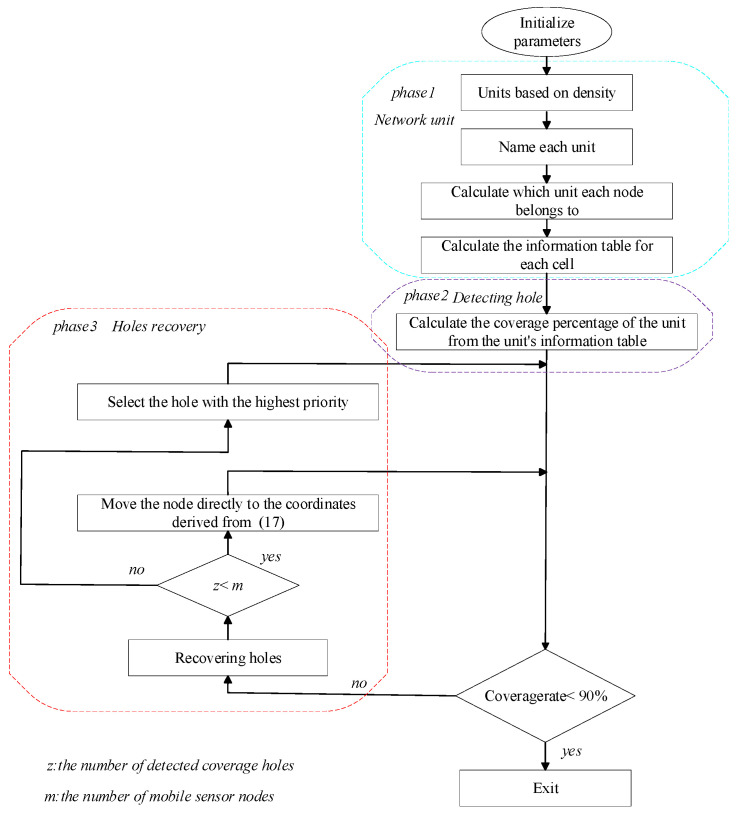
Basic framework.

The proposed method is designed to address the issue of detecting and recovering coverage holes in wireless sensor networks through a three-stage process. In the initial phase, the network is partitioned into a varying number of equally sized units, depending on the density of the nodes. Then an agent is selected for each unit based on two conditions: the distance of the node from the center point of the unit and the remaining energy of the node. After that, the information is transmitted through the agent node and when the energy of the agent node is less than 10%, the agent node is reselected. In the second stage of the method, each unit has an information table, and the percentage of nodes covering each unit can be calculated more accurately based on the information table. When the percentage of the unit is less than 90%, we consider that the unit has coverage holes. After that, it is necessary to fix the coverage holes. In the third stage, when the coverage holes are detected, we use the average value of the key perceptual interactions as the initial value of the global optimal point of the PSO, and then we send the mobile nodes to repair the holes. Meanwhile, we consider three factors when fixing the holes, including the distance of the BS from the hole unit, the coverage ratio of the hole, and the overlap of the mobile node with other nodes. In our work, the basic framework of the algorithm in this paper is shown in Figure 4. The following section describes the algorithm of this paper in detail.

### 4.1. Network Unit

In this phase, the network environment is unitized based on its density. To achieve optimal load distribution, the number of network units dynamically adjusts to reflect the network’s density, with denser networks resulting in a greater number of uniform-sized units. Initially, the network density is determined using Formula (5), which considers both the number of nodes and the network’s area.

(5) $$Density=\frac{n}{L^{2}}$$

In (5) *n* is the number of static sensor nodes and *L* is the width of the monitoring environment. Based on (6), the network is divided into different numbers of units according to the network density.

(6) $$C=\left\{\begin{array}{l} 2^{2},\;if(0\leq Density\leq 0.001)\\ 3^{2},\;if\left(0.001\leq Density\leq 0.01\right)\\ 4^{2},\;if(0.01\leq Density\leq 0.05)\\ 5^{2},\;if(0.05\leq Density\leq 0.1)\\ 6^{2},\;if(0.1\leq Density\leq 0.15)\\ 7^{2},\;if(0.15\leq Density\leq 0.2)\\ \ldots \\ \ldots \\ \ldots \end{array}\right.$$

where *C* is the number of divided units. The size of each unit is calculated according to (7).

(7) $$Cellsize=\frac{L}{\sqrt{C}}$$

**Figure 5:**
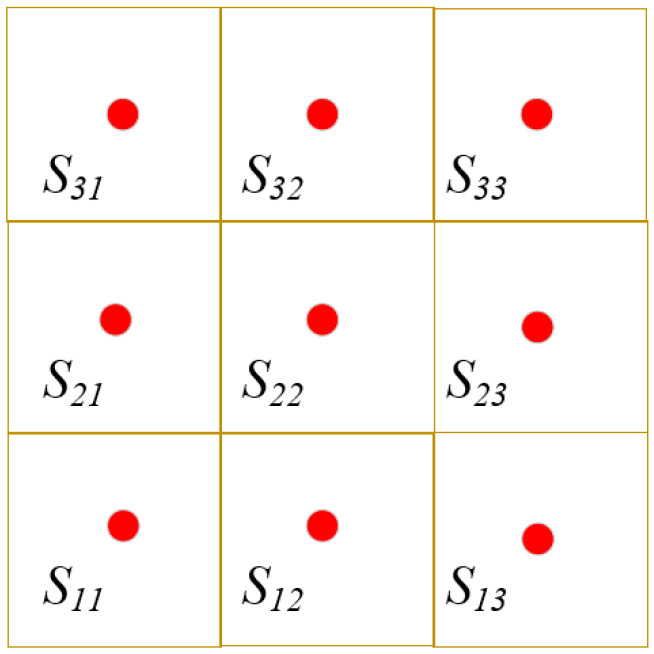
Unit naming.

After that the unitized unit is named as shown in Figure 5.

The *A_ij_* represent a unit, where *i* signifies the row position of the unit within a grid, and *j* represents its column position. Meanwhile, (*x*_0_, *y*_0_) represents the location of the base station.

According to (8), each individual node can determine the unique unit number in which it is located.

(8) $$\left\{\begin{array}{c} i=\left\lceil \frac{y_{s}}{Cellsize}\right\rceil \\ j=\left\lceil \frac{x_{s}}{Cellsize}\right\rceil \end{array}\right.$$

where (*x_s_*, *y_s_*) is the position of UAV and *Cellsize* is the size of the unit.

In each unit, the node with the most remaining energy and closest to the unit center is selected as the unit agent. It is assumed that each unit has a unit centroid, such as shown in Figure 5.

The coordinates of the center point of each unit are calculated according to (9).

(9) $$\;\left(\left[x_{0}+\left(j-\frac{1}{2}\right)\;*\;Cellsize\right],\left[y_{0}+\left(i-\frac{1}{2}\right)\;*\;Cellsize\right]\right)$$

where (*x*_0_, *y*_0_) represents the location of the base station, *i* represents the horizontal coordinate of the node, *j* represents the vertical coordinate of the node, and *Cellsize* represents the size of the unit.

**Figure 6:**
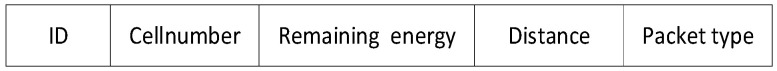
Hello message.

All of the nodes send a hello message containing node *ID*, *Cell-number*, *Remaining energy*, *Distance*, and *Packet type* to other neighboring nodes in the same unit as shown in Figure 6. The *ID* represents the node number, *Cellnumber* represents the unit number where the node is located, *Remaining energy* represents the remaining energy of the node, *Distance* represents the distance from the node to the center point of the unit, and *Packet type* represents the packet type, which initially has a value of 0.

Then, each node is compared with its neighbors. The optimal unit agent is selected and it will send a packet containing its *ID* and *Cellnumber* to its co-unit node and declare itself as a unit agent. If the value of *Packet type* changes to 1, it means that the node is a unit agent.

When the energy level of a unit agent drops below 10%, an agent notification packet is transmitted to its co-unit node, and the process of selecting a new unit agent for that unit is repeated. This mechanism ensures that the unit agent is changed as needed to function properly and fulfill its responsibilities effectively. As depicted in Figure 6, in addition to the *Cellnumber* and *ID*, the *Packet type* is also included in the transmission. If the *Packet type* changes to 0, it indicates that the unit agent has been reselected.

Algorithm 1 gives the pseudo-code for the first stage.
**Algorithm 1.** Network unit
**Input**: *i*: row number, *j*: column number, *L*: length of the network, *C*: number of network units, *n*: number of nodes**Output**: *C*, *A_ij_*
1: The network density is calculated
2: Density = *n/L^2^*
3: The number of units is calculated according to the Density
4: Each unit is named as *A_ij_*
5: The side size of each unit in the network is calculated by $Cellsize=\frac{L}{\sqrt{C}}$
6: The base station notifies (*x*_0_, *y*_0_) and *Cellsize* to all network nodes
7: **for** (*s* = 1; *s* ≤ *n*; *s*++) **do**
8:       Identifying unit number in which it is located for nodes by $\left\{\begin{array}{c} i=\frac{y_{s}}{Cellsize}\\ j=\frac{x_{s}}{Cellsize} \end{array}\right.$
9:      Node s sends a hello packet to the neighbor nodes
10: **end for**
11: **if** (The node has the most remaining energy and closest to the center of the unit) then
12:    Sends a packet and declares itself a unit agent
13: **else**
14:    Waits to receive the unit agent notification packet
15: **end if**

### 4.2. Detecting Holes

In this stage, each unit is discretized into *k* ∗ *k* pixel points and the set of pixel points is *U_i_* = (*x_i_*, *y_i_*), and *i* = 1, 2, 3, …, *k*. Each unit has an information table to calculate the proportion of each unit covered by nodes. The quantity of rows present in this table corresponds directly to the total number of pixels encompassed by the unit, referred to as *k*. Similarly, the count of columns within this table matches the number of nodes contained within the same unit.

**Figure 7:**
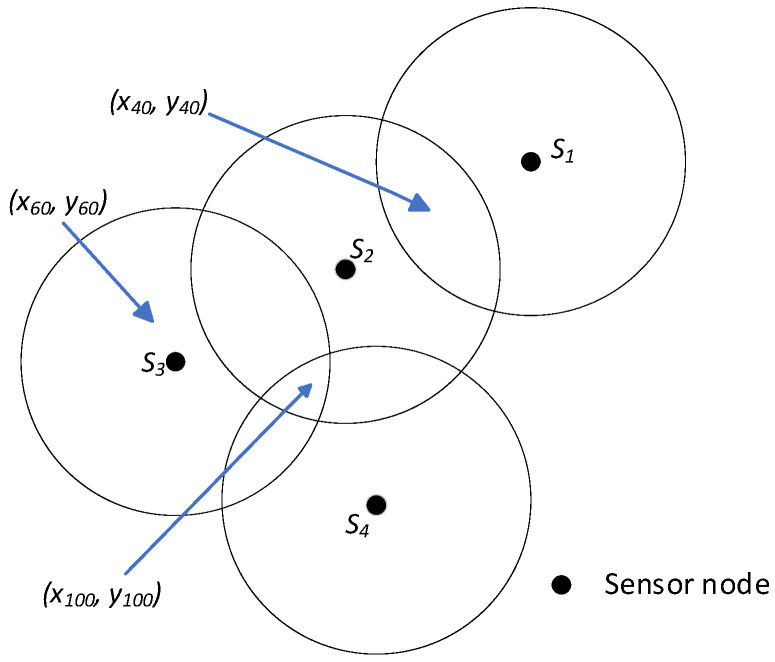
Overlapping pixel points about unit *A*_11_.

If a pixel is covered by at least one neighboring node, the value in its corresponding *CONO* column is set to 1. The number of rows in each information table is consistent, while the number of columns varies depending on the number of nodes within each unit. Initially, all entries in the information table are set to 0. When a pixel is covered by a node, the table entry corresponding to that pixel is updated to 1. Table 1 shows a complete example of an information table for unit *A*_11_, according to Figure 7.

As shown in Figure 7, (*x*_40_, *y*_40_) is covered by two nodes *S*_1_ and *S*_2_, and (*x*_100_, *y*_100_) is covered by three nodes *S*_2_, *S*_3_, and *S*_4_. Therefore, the (*x*_40_, *y*_40_) entries in the columns of nodes *S*_1_ and *S*_2_ in Table 1 are 1. The (*x*_100_, *y*_100_) entries in the columns of nodes *S*_2_, *S*_3_, and *S*_4_ are 1. (*x*_1_, *y*_1_), (*x_k_*_−1_, *y_k_*_−1_), and (*x_k_*, *y_k_*) are not covered by any node. Hence, the table entries for these pixels are all 0 in Table 1. Additional pixels are also evaluated using this same method.

The number of pixels per unit is calculated based on the length and width of the unit, based on (10).

(10) $$\;{Cell}_{pixel}={\left(Cellsize\;*\;30\right)}^{2}$$

The proportion of each unit covered by nodes is calculated according to (11).

(11) $${Coveragerate}_{sij}=\frac{CoNO}{{Cell}_{pixel}}$$

If the coverage of the unit is greater than or equal to 90 per cent, the unit does not have a coverage hole. Otherwise, the unit has a coverage gap.

**Figure 8:**
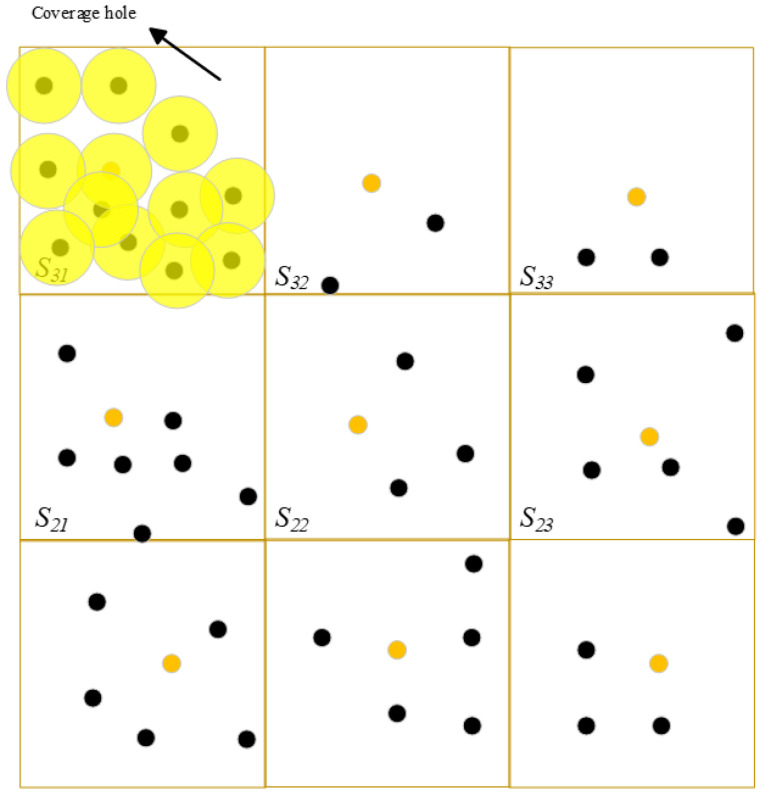
Coverage hole in a unit.

Figure 8 shows a cell of the network after the holes are detected.

Algorithm 2 gives the pseudo-code for the second stage.
**Algorithm 2.** Detecting Holes**Input:** *k*: number of pixels covered by the unit, *n*: number of nodes, *d*: Euclidean distance, *C*: number of network units, *R_s_*: sensing radius **Output:** *Coveragerate_sij_*
1: *Information = 0*
2: **for** (*s* = 1; s ≤ *C*; *s*++) **do**
3:      **for** (*k* = 1; *d* ≤ *R_s_*; *k*++) **do**
4:         All the entries of a row k in the Info table are OR together and recorded in *CoNO* column
5:      **end for**
6:      Calculate the coverage of each unit (*Coveragerate_sij_*)
7: **end for**
8: **for** (*s* = 1; *s* ≤ *C*; *s*++) **do**
9:      **if** (*Coveragerate_sij_* < 90%) **then**
10:      This unit has holes.
11:    **else**
12:        There are no holes in this unit.
13: **end if**

### 4.3. Hole Recovery

**Figure 9:**
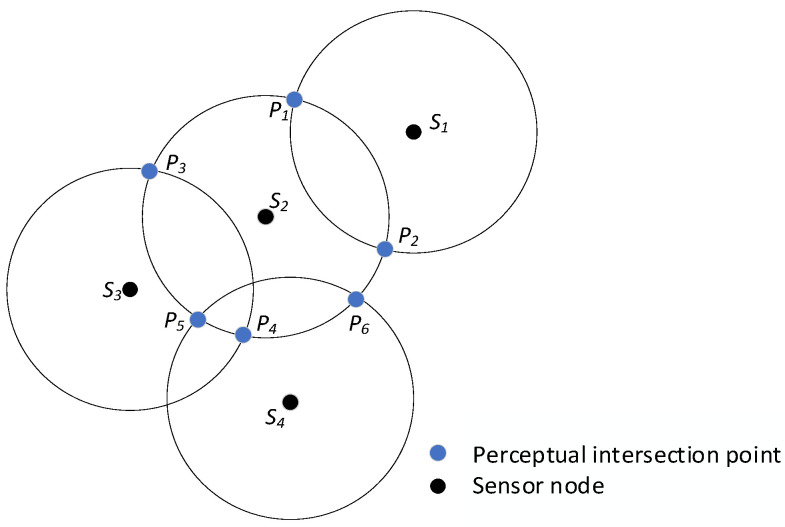
Node distribution diagram for unit *A*_11_.

In this phase, we need to first find the key perceptual intersection of all nodes within each unit. For example, Figure 9 shows the distribution of nodes in unit *A*_11_. In Figure 9, node *S*_1_ intersects its neighbor node *S*_2_ at *P*_1_ and *P*_2_, and the set {*P*_1_, *P*_2_} are no longer covered by any other nodes. Therefore, the set {*P*_1_, *P*_2_} is the key perceptual intersection of node *S*_1_. Node *S*_2_ has three neighboring nodes, *S*_1_, *S*_3_, and *S*_4_. The intersection of *S*_2_ with *S*_1_ is *P*_1_, *P*_2_, and *P*_1_, *P*_2_ are no longer covered by other nodes, so *P*_1_, *P*_2_ are part of the key perceptual intersection set of *S*_2_. The intersection of *S*_2_ with *S*_3_ is *P*_3_, *P*_4_, but this intersection of *P*_4_ is covered by *S*_4_, so *P*_4_ is not a key perceptual intersection set of *S*_2_, and similarly, *P*_5_ is also is not a key perceptual intersection of *S*_2_. The final result is that the set {*P*_1_, *P*_2_, *P*_3_, *P*_6_} is the key perceptual intersection of *S*_2_.

With this method, the key perceptual intersections of all nodes in the unit are derived. After that, the key perception intersection of all nodes in the unit is derived, but the exact location coordinates of all KIPs need to be known. The key perceived intersections *P*_1_, *P*_2_ of node *S_i_* with its neighbor node *S_j_* are calculated as follows:

(12) $$x_{p1}=\delta +\frac{\gamma \left(y_{j}-y_{i}\right)}{d_{i,j}},y_{p1}=\eta +\frac{\gamma \left(x_{j}-x_{i}\right)}{d_{i,j}}$$

(13) $$\;x_{p2}=\delta -\frac{\gamma \left(y_{j}-y_{i}\right)}{d_{i,j}},y_{p2}=\eta +\frac{\gamma \left(x_{j}-x_{i}\right)}{d_{i,j}}$$

where (*x_i_*, *y_i_*), (*x_j_*, *y_j_*) are the position coordinates of nodes *S_i_*, *S_j_*, respectively, *d_i_*_,*j*_ denotes the Euclidean distance between node *S_i_* and node *S_j_*, (*x_p_*_1_, *y_p_*_1_) are the coordinates of intersection point *P*_1_, and (*x_p_*_2_, *y_p_*_2_) are the coordinates of intersection point *P*_2_. *δ*, *η*, and *γ* are calculated using the following three equations:

(14) $$\;\delta =x_{i}+\left(\frac{{R^{2}}_{si}-{R^{2}}_{sj}+{d^{2}}_{ij}}{2d_{ij}}\right)\left(\frac{x_{j}-x_{i}}{d_{ij}}\right)$$

(15) $$\;\eta =y_{i}+\left(\frac{{R^{2}}_{si}-{R^{2}}_{sj}+{d^{2}}_{ij}}{2d_{ij}}\right)\left(\frac{y_{j}-y_{i}}{d_{ij}}\right)$$

(16) $$\gamma =\sqrt{{R^{2}}_{si}-\left(\frac{{R^{2}}_{si}-{R^{2}}_{sj}+{d^{2}}_{ij}}{2d_{ij}}\right)}$$

where *R_si_*, *R_sj_* represent the perceived radius of node *S_i_* and node *S_j_*.

The coordinates of the geometric center of the hole shape are then calculated using (17).

(17) $$\bar{X_{i}}=\frac{\sum_{i=1}^{n}x_{i}}{n},\bar{Y_{i}}=\frac{\sum_{i=1}^{n}y_{i}}{n}$$

where (*x_i_*, *y_i_*) are the coordinates of each key sensing point. *n* represents the number of key sensing intersections in this unit.

**Figure 10:**
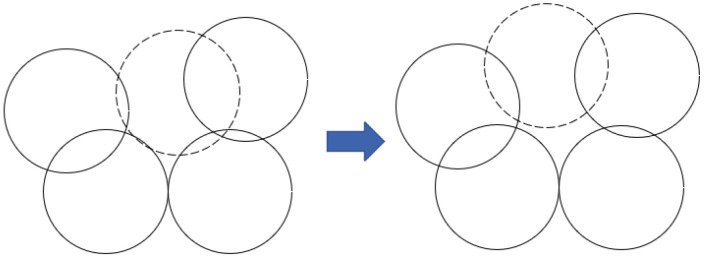
New location of mobile node.

Then, PSO is used to determine the most necessary holes covered by using mobile nodes. There are m mobile nodes available for coverage hole recovery, and the number of detected coverage holes is *z*. When *z* ≤ *m*, the mobile nodes are directly sent to the coordinates derived from (17) for hole recovery, when the mobile nodes take into account the overlap rate with other nodes. As shown in Figure 10, the dashed position on the right side is the new position of the mobile node derived by the algorithm in this paper. When *z* > *m*, the highest priority hole is selected according to PSO. The steps in this phase of the proposed method are outlined as follows:

Initialization: In this paper, *g* particles are used as the initial population. Each particle is considered to cover the response of the hole.

Fitness function: In (18), the fitness function of the method in this paper is shown.

(18) $$\begin{array}{c} \;Fit=a\;*\;\left(1-\frac{D_{bs,h}}{L}\right)+b\;*\;\left(1-{Coveragerate}_{sij}\right)\\ +\;c\;*\;\left(1-{Overlaprate}_{sn}\right) \end{array}$$

where *D_bs_*_,*h*_ denotes the distance from the base station to the hole unit, calculated based on the Euclidean distance. *Coveragerate_sij_* is the coverage of the unit *S_ij_*. *Overlaprate_sn_* represents the overlap rate between the mobile node *S_n_* and other nodes. *L* is the network width. *a*, *b*, and *c* are the influence coefficients, which take values in the range of 0~1 and *a* + *b* + *c* = 1.

In this step, each particle is evaluated according to the fitness function in (18). The solution with the highest fitness function value is designated as *T*.

End condition: In this paper, the termination condition is set to reach a simulation time of 150 s. If the end condition is satisfied, *T* is considered to be the answer; otherwise, the step continues.

Position update: The position of the particle is updated in accordance with (19).

(19) $${x_{id}}^{k+1}={x_{id}}^{k}+{v_{id}}^{k}$$

In (19), ${x_{id}}^{k+1}$-the position vector of particle i in the dth dimension in the *k* + 1th iteration. ${x_{id}}^{k}$-the velocity vector of particle *i* in the *d_th_* dimension in the *k_th_* iteration. ${v_{id}}^{k}$-velocity vector of particle *i* in the *d_th_* dimension in the *k_th_* iteration.

Then, the velocity of the particle is updated according to (20).

(20) $$\begin{array}{c} \;{v_{id}}^{k+1}=\omega \;{v_{id}}^{k}+c_{1}r_{1}\left({p_{id,pbest}}^{k}-{x_{id}}^{k}\right)\\ +\;c_{2}r_{2}\left({p_{d,gbest}}^{k}-{x_{id}}^{k}\right) \end{array}$$

In (20), *N*—particle swarm size; *i*—particle serial number, *i* = 1, 2, …, *N*; *D*—particle dimension; *d*—particle dimension serial number, *d* = 1, 2, …, *D*; *k*—number of iterations; *w*—inertia weight; *c*_1_—individual learning factor; *c*_2_—population learning factor; and *r*_1_, *r*_2_—random numbers in the interval [0, 1] to increase the randomness of the search.

Return to the evaluation step: After the position update, the new particles are evaluated. These steps are iteratively repeated until the stopping condition is met.

Algorithm 3 gives the pseudo-code for the third stage.
**Algorithm 3.** Hole Recovery**Input:** *z*: number of coverage hole, *m*: number of mobile nodes, *n*: the number of key perceptual point**Output:** New coverage of Unit *S_ij_*
1: *Coverageratesij* < 90%
2: **if**
*z* ≤ *m*
**then**
3:     **for** (*z* = 1; *z* ≤ *m*; *z*++) **do**
4:         The mobile node is directly sent to the coordinates for hole recovery by $\bar{X_{i}}=\frac{\sum_{i=1}^{n}x_{i}}{n},\bar{Y_{i}}=\frac{\sum_{i=1}^{n}y_{i}}{n}$.
5:   **else**
6:        **for** (*z* = 1; *z* > *m*; *z*++) **do**
        the highest priority hole is selected according to Particle Swarm Optimization algorithm
7:          The priority of holes depends on three parameters, including *D_bs_*_,*h*_, *Coveragerate_sij_*, and *Overlaprate_sn_*.
8:        **end for**
9: **end if**

## 5. Simulation Experiment and Result Analysis

**Figure 11:**
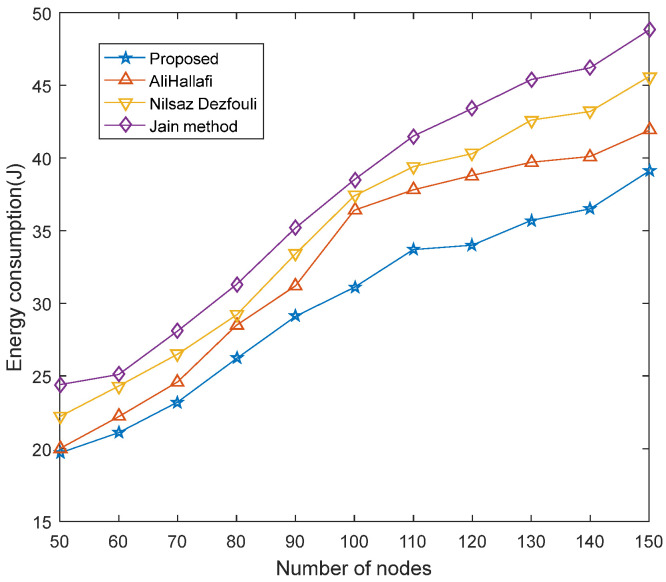
Energy consumption with different numbers of nodes.

**Figure 12:**
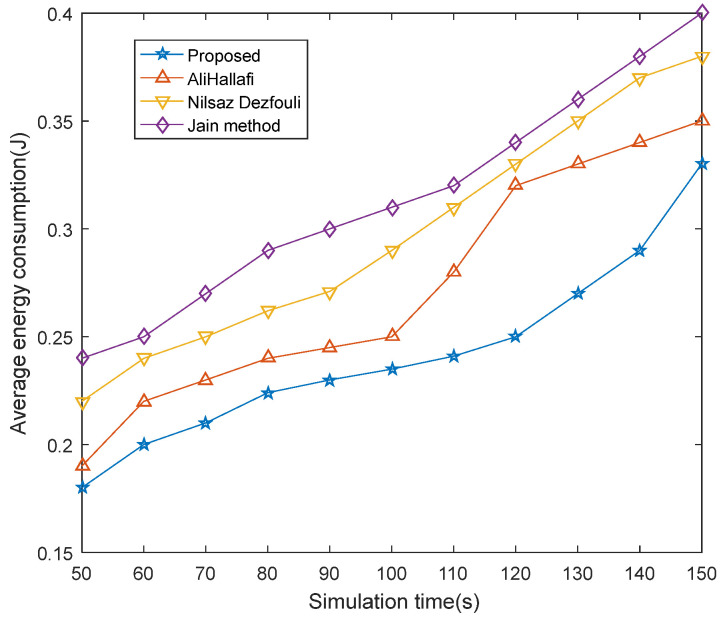
Average energy consumption at different simulation times.

**Figure 13:**
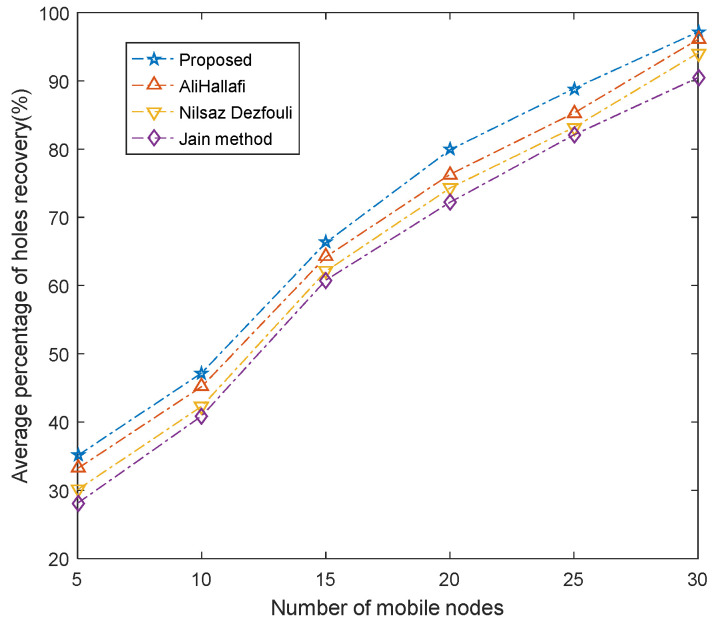
Average percentage of hole recovery for various numbers of mobile nodes.

**Figure 14:**
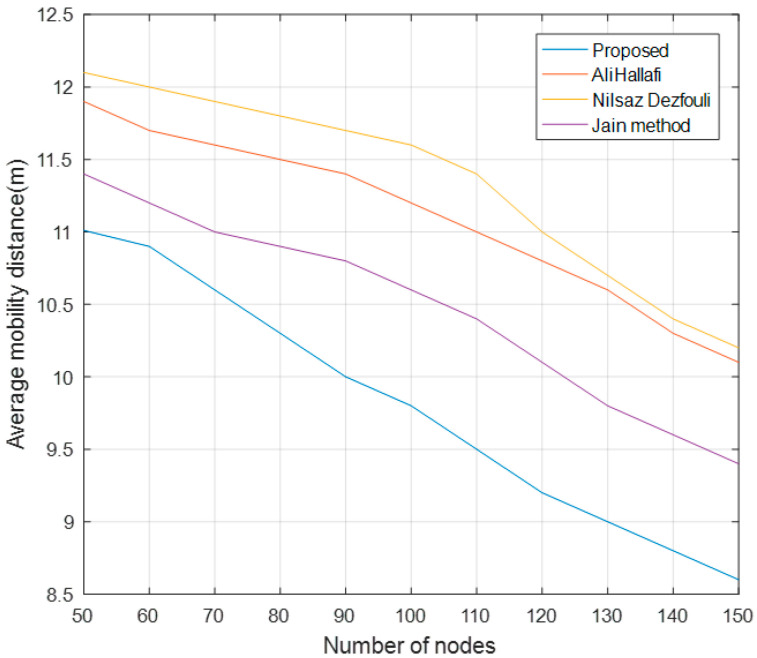
Average mobility distance of mobile nodes with different numbers of nodes.

**Figure 15:**
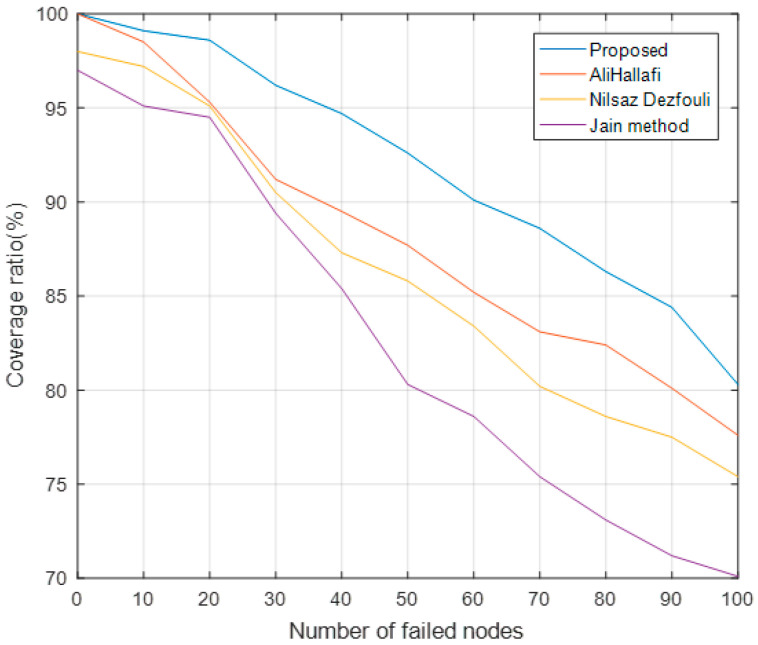
Coverage ratio with different number of failed nodes.

**Figure 16:**
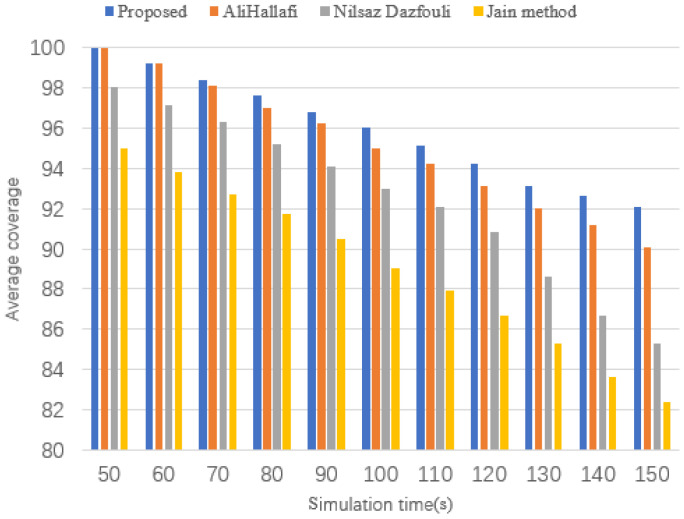
Average coverage at different simulation times.

**Figure 17:**
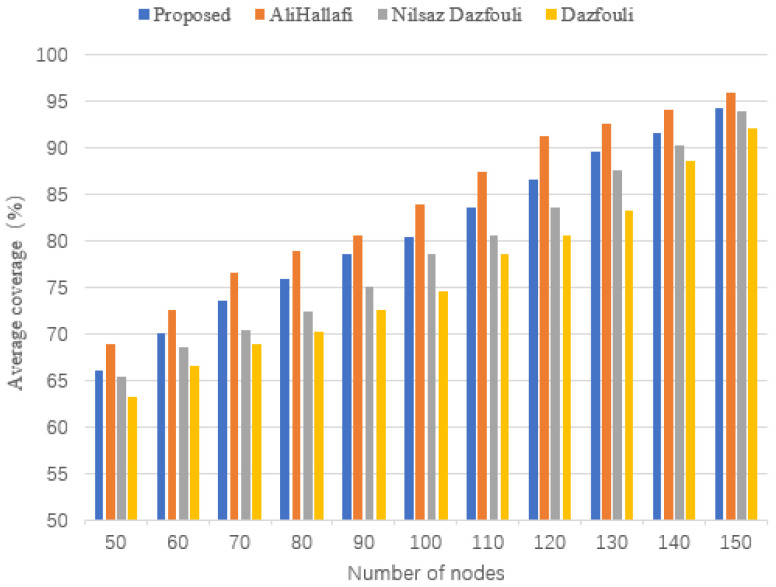
Average coverage with different numbers of nodes.

In this chapter, pycharm is used to construct a 2D simulation scenario to verify the performance of the algorithms in this paper. A total of 150 static nodes are randomly distributed in a 2D area of 200 m × 200 m, and 30 mobile nodes are located near the base station. Then, the AliHallafi [9], Nilsaz Dazfouli [15], and Jain methods [30] and this paper’s algorithm are used, and the algorithm is verified. The specific parameters used for the simulation are shown in Table 2. The simulation results and analysis are shown in Figure 11, Figure 12, Figure 13, Figure 14, Figure 15, Figure 16 and Figure 17.

### 5.1. Comparison of Energy Consumption

Figure 11 shows a comparison of the energy consumed when running the AliHallafi, Nilsaz Dazfouli, and Jain methods and proposed algorithm in this paper with different numbers of sensor nodes. In WSNs, the energy consumption mainly comes from sensing, the movement of mobile nodes, and data transmission between nodes and nodes. In this paper, most of the energy consumption comes from the movement of mobile nodes. The algorithm proposed in this paper unitizes the network which reduces the energy consumption and the network unitization is performed based on the density of the nodes which will make the energy consumption more balanced. Thus, the algorithm of this paper reduces energy consumption by 6.68% compared with AliHallafi, 16.22% compared with the Nilsaz Dazfouli, and 19.87% compared with the Jain method.

### 5.2. Comparison of Average Energy Consumption

Figure 12 presents a comparative analysis of average energy consumption for running the AliHallafi, Nilsaz Dazfouli, and Jain methods and the proposed algorithm in this paper for different scenarios of simulation time. In this paper, the fitness function in the particle swarm optimization algorithm is used to select the holes to be repaired by the mobile node. The parameters used to select the hole to be patched are the distance from the hole to the base station, the size of the hole, and the overlap rate of the mobile node with other nodes, which allows the mobile node to move a shorter distance and cover a larger area. Thus, the average energy consumption of this paper’s method is 5.71% less than the AliHallafi, 13.15% less than the Nilsaz Dazfouli, and 17.5% less than the Jain method with different simulation times.

### 5.3. Average Percentage of Holes Repaired

Figure 13 shows the comparison of the average percentage of holes repaired by running the AliHallafi, Nilsaz Dazfouli, and Jain methods and the proposed algorithm in this paper for different numbers of mobile nodes. The percentage of holes fixed increases as the amounts of mobile nodes continues to increase. In this paper, the coverage area of each unit by nodes can be calculated more accurately, and the hole detection accuracy is high, so better hole recovery decisions can be made. When a coverage hole is detected in a unit, the mobile node is sent to the hole unit. Therefore, for different numbers of mobile nodes, the average hole recovery rate of our algorithm is 2.16% higher than the AliHallafi, 4.29% higher than the Nilsaz Dazfouli, and 7.42% higher than the Jain method.

### 5.4. Average Mobility Distance of Mobile Nodes

Figure 14 shows the comparison of the average mobility distance of mobile nodes running the AliHallafi, Nilsaz Dazfouli, Jain methods and this paper’s algorithm with different numbers of nodes. The algorithm in this paper takes the average value of the key perceptual intersection as the initial value of the global optimum in the particle swarm optimization algorithm and sets a more optimal fitness function when selecting the mobile nodes, which reduces the travel distance of the mobile nodes. As a result, the average traveling distance of this paper’s algorithm is reduced by 16.5% compared with the Nilsaz Dazfouli, 14.85% compared with AliHallafi, and 8.51% compared with the Jain method for different numbers of nodes.

### 5.5. Coverage Ratio with Different Number of Failed Nodes

Figure 15 shows the coverage ratio of running the AliHallafi, Nilsaz Dazfouli, and Jain methods and this paper’s algorithm with different numbers of failed sensors. The algorithm in this paper can calculate the location of the coverage holes more accurately and will repair the coverage holes according to the fitness function. The AliHallafi and Nilsaz Dazfouli methods can wake up the nodes according to the overlap rate of the nodes but they dispatch the nodes to move them to repair the coverage holes with a simple fitness function. Therefore, as the number of failed nodes increases, the coverage ratio of our algorithm is 3.36% higher than the AliHallafi, 6.10% higher than the Nilsaz Dazfouli, and 12.71% higher than the Jain method.

### 5.6. Average Coverage at Different Simulation Times

Figure 16 shows the average coverage for running the AliHallafi, Nilsaz Dazfouli, and Jain methods and the proposed algorithm in this paper for different scenarios of simulation time. The algorithm in this article uses the network unit to reduce energy consumption, while combining the particle swarm optimization algorithm with KIP also reduces energy consumption. Therefore, with an increase in simulation time, the average coverage of our algorithm is 2.17% higher than the AliHallafi, 7.38% higher than the Nilsaz Dazfouli, and 10.53% higher than the Jain method.

### 5.7. Average Coverage

Figure 17 shows the comparison of the average coverage of running the AliHallafi, Nilsaz Dazfouli, and Jain methods and this paper’s algorithm with different numbers of sensor nodes. The average coverage of this paper is 2.41% higher than the Nilsaz Dazfouli and 4.15% higher than the Jain method for different number of nodes but the average coverage under the AliHallafi algorithm is higher than the average coverage of this paper’s algorithm. This is mainly because this paper aims to reduce the energy consumption of the nodes, increase the percentage of fixing holes, and reduce the distance traveled by the mobile sensor nodes.

## 6. Conclusions

This paper proposes a method for hole detection and hole recovery based on the key perceptual intersection for hybrid wireless sensor networks. This method first divides the network into equally sized units, and then selects a representative node for each unit, called an agent. Then, the proportion of each unit covered by nodes is accurately calculated and holes are detected. Finally, the holes are recovered using the average of the key perceptual intersection as the initial value of the global optimal point of the particle swarm optimization algorithm. The simulation experiments illustrate that the proposed algorithm reduces the network energy consumption, reduces the traveling distance of the mobile nodes, and increases the percentage of network holes that are repaired. These improvements collectively enhance the overall performance of the algorithm, specifically manifested in the following ways: network modularization significantly reduces node energy consumption; increasing the proportion of coverage vulnerabilities repaired enhances network coverage effectiveness; and reducing node movement distance improves algorithm efficiency. In future, we will improve the average coverage under different numbers of nodes while maintaining the advantages of our algorithm.

## Figures and Tables

**Table 1:** Information table for unit *A*_11_.

Coordinate	*S* *_1_*	*S* *_2_*	*S* *_3_*	*S* *_4_*	*S* *_5_*	…	*S* *_n_*	CoNO
**(*x*_1_**, ***y*_1_)**	**0**	**0**	**0**	**0**	**0**		**0**	**0**
**…**								
**(*x*** **_40_** **, *y*_40_** **)**	**1**	**1**	**0**	**0**	**0**		**0**	**1**
**…**								
**(*x*_1_** **_00_** **, *y*_1_** **_00_** **)**	**0**	**1**	**1**	**1**	**0**		**0**	**1**
**…**								
**(*x*** ***_k_*** **_−_** **_1_** **, *y*** ***_k_*** **_−_** **_1_** **)**	**0**	**0**	**0**	**0**	**0**		**0**	**0**
**(*x*** ***_k_*** **, *y*** ***_k_*** **)**	**0**	**0**	**0**	**0**	**0**		**0**	**0**

**Table 2:** Simulation parameters.

Symbol	Attribute	Value
*O*	monitoring area	200 ∗ 200 m^2^
*Deploy*	deployment method	Random
*L*	length of the network	200
*Si*	a sensor node	180
*Aij*	represents a unit	
*C*	number of units	Depends on the number of nodes
*Rs*	sensor i sensing radius	30 m
*Rc*	sensor i communication radius	60 m
*Vi*	movement speed of sensor i	20 m/s
*n*	number of static sensor nodes	150
*m*	number of mobile sensor nodes	30
*Em*	initial energy of the mobile node	5 J
*Ec*	mobile energy consumption	0.1 J/m
*SIM*	simulation time	150 s

## Data Availability

The data are available from the corresponding author on reasonable request.
